# Digital economy and institutional dynamics: striving for equitable public service in a digitally transformed era

**DOI:** 10.3389/fpubh.2024.1330044

**Published:** 2024-03-21

**Authors:** Yuwen Lyu, Junxian Xie, Xulei Meng, Xiang Wang

**Affiliations:** ^1^Institute of Humanities and Social Sciences, Guangzhou Medical University, Guangzhou, China; ^2^School of Culture and Creativity, Beijing Normal University - Hong Kong Baptist University United International College, Zhuhai, China; ^3^International Department of the Affiliated High School of South China Normal University, Guangzhou, China; ^4^Zhou Enlai School of Government, Nankai University, Tianjin, China

**Keywords:** digital economy, institutional environment, equalization of public service provision, social equity, common prosperity

## Abstract

**Background:**

The rapid emergence of China’s digital economy has sparked profound interest in the complex interplay between digitalization and the provision of public services. This study aims to delve deeper into how the development of the digital economy impacts the level of equalization in public service delivery and evaluates whether institutional factors can moderate this transformation. Against the backdrop of pursuing “common prosperity,” this research provides valuable guidance for policymaking and strategic planning. It ensures that the ascent of the digital economy not only elevates the standards of public services but also fosters their equitable distribution, thereby advancing the cause of social equity.

**Methodology:**

The study utilized the System Generalized Method of Moments (GMM) model along with longitudinal trend data spanning from 2009 to 2018. This approach facilitated an in-depth analysis of the relationship between the digital economy and the level of equalization in public service delivery. The application of this model provided deeper insights into the impact of the digital economy on public service equalization and the identification of underlying mechanisms.

**Findings:**

This study reveals a complex paradox that the digital economy is exacerbating regional disparities in the provision of basic public services. Furthermore, the research underscores the pivotal role of institutional environments in mitigating the adverse effects of the digital economy on public service provision. By examining the interplay between digital economy growth and institutional frameworks, the study suggests that adaptable and robust institutions are essential for harnessing the digital economy’s benefits while minimizing its potential drawbacks.

**Conclusion:**

In conclusion, the findings from this study offer substantial insights into the dual impact of the digital economy on public service provision, enriching the ongoing discourse on digital transformation and social equity. The research underscores the significance of strategic policy reforms and institutional adjustments to harness the transformative power of the digital economy, promoting equitable access to public services and advancing the goal of “common prosperity” in the digital age.

## 1 Introduction

The digital economy’s rise, marked by the deep integration of digital technologies into economic processes, has catalyzed significant global transformations. This new economic paradigm, driven by digital networks and processes, spans a range of activities, from e-commerce to cloud computing (1). Concurrently, public services, encompassing essential government-provided services such as healthcare and education, are pivotal to societal welfare (2). The convergence of the digital economy with public service delivery presents a complex landscape of opportunities and challenges, especially in terms of adapting and integrating digital advancements (3, 4).

This study seeks to address a notable gap in existing research by exploring the dual impact of the digital economy on public service provision, particularly within the context of the institutional environment. Previous studies have primarily focused on either the positive or negative aspects of digital transformation in public services, often overlooking the nuanced interplay between these factors (5, 6). Our research builds on this foundation by delving into how the institutional environment, characterized by its rules, norms, and regulations (7, 8), mediates the influence of digital technologies on public services. We specifically address the critical issue of the digital divide and its impact on service equality (9), an area that remains underexplored in current literature.

The contribution of this study lies in its in-depth analysis of how the digital economy interacts with the institutional environment to affect public service provision. By providing a comprehensive exploration of these relationships, our research offers valuable insights that extend beyond the existing theoretical frameworks. It significantly contributes to the academic discourse on digital transformation in public services by highlighting the role of institutional factors in shaping these outcomes. This approach not only enhances the theoretical understanding of the digital economy’s dynamics but also provides practical implications for improving public service delivery in the digital age.

Furthermore, our study stands out for its holistic examination of the interactions between digital technologies, institutional factors, and public service outcomes. It aims to guide effective policy and strategic decisions for government and digital sector stakeholders, thereby filling a critical void in the current academic landscape.

The paper is structured to first present a comprehensive literature review, followed by the formulation of research hypotheses, a detailed description of our methodology, and a discussion of the findings. We conclude by outlining the policy implications of these findings and suggesting directions for future research.

## 2 Literature review and hypotheses

### 2.1 Literature review

#### 2.1.1 Impact of the digital economy on public services

The digital economy, characterized by the integration of digital technology in economic activities (10–12), has significantly reshaped public service delivery. Its influence extends from operational efficiencies to service accessibility (13, 14).

Current scholars demonstrate how digitalization facilitates more efficient, transparent, and citizen-centric public services (15, 16). However, as Margetts and Dunleavy caution, without adequate infrastructure and policy support, the digital economy can exacerbate existing service delivery inequalities (17).

#### 2.1.2 The role of institutional frameworks

The institutional environment plays a critical role in how digital transformation impacts public services. Institutions, encompassing regulations, norms, and policies, determine the successful integration of digital technologies in public sectors (18, 19). For instance, Sazanova and Kuznetsov highlights that supportive institutional structures are essential for leveraging digital technologies’ benefits and mitigating potential inequalities (20). This is further supported by research discussing the critical role of institutional rules and governance in influencing innovation and service delivery within the public sector (21).

#### 2.1.3 Addressing the digital divide

The digital divide poses a significant challenge in the digitalization of public services, extending beyond mere access issues to encompass disparities in digital literacy and utilization (22). It has been highlighted that without effectively addressing these disparities, the digital economy may inadvertently exacerbate gaps in service provision, disproportionately impacting marginalized and underserved communities (23). This underscores the importance of comprehensive strategies aimed at ensuring equitable digital participation and access for all sectors of society.

### 2.2 Hypotheses

Based on this comprehensive literature review, the study proposes two hypotheses:

*H1*: The development of digital economy is negatively correlated with the equalization of public services. Although prevailing studies indicate that digitalization has the potential to enhance the availability and caliber of public services, the effects are not uniformly dispersed among different regions. This uneven impact risks aggravating existing geographical inequalities in the provision of public services.

*H2*: The institutional environment moderates the relationship between the digital economy and the equalization of public service provision. This is grounded in research indicating that the impact of digital transformation on public services is significantly influenced by institutional factors, including policy frameworks and governance structures.

In the summary, this study aims to bridge the gap in understanding the complex relationship between the digital economy, institutional frameworks, and public service provision. It seeks to offer insights into how digital advancements and institutional dynamics collectively shape public service outcomes, contributing to a nuanced perspective in this evolving field.

## 3 Methodology, variables, and data

### 3.1 Model construction

This study adopts a dynamic panel data model to investigate the effects of digital economy development on the equalization of public service provision. This approach allows for the accounting of temporal dynamics and potential endogeneity inherent in the relationships being studied. Specifically, the System Generalized Method of Moments (SYS-GMM) is utilized, a method widely recognized for its robustness in handling the challenges posed by dynamic panel data (24, 25).

The SYS-GMM methodology is particularly effective at dealing with unobservable individual effects and endogeneity by employing lagged values of the variables as instruments. The choice of SYS-GMM is informed by its success in previous research, such as the comprehensive discussion by Roodman (26) on the application of GMM in economic research, and Bond (27), who highlights the efficiency gains of SYS-GMM over other estimators in the context of dynamic panels.

The model constructed, as specified by Equation (1):


(1) $$BPS:equa_{\mathrm{it}}=\alpha +\mathrm{wBPS}:equa_{\mathrm{it}-j}+\gamma DE_{\mathrm{it}}+\delta lnX_{\mathrm{it}}+\varepsilon_{i}$$

$BPS:equa_{\mathrm{it}}$ is the explained variable, the equalization of public services in each region; $DE_{\mathrm{it}}$ represents the level of digital economy development in year t of province i; $BPS:equa_{\mathrm{it}-j}$ is the lag term of equalization degree; *X* is a set of control variables, by taking the logarithm method to eliminate the effect of heteroscedasticity ;w represents the matrix of coefficients; $\varepsilon_{\mathrm{it}}$ is the perturbation term; and α represents the possible individual effects of the model.


(2) $$\begin{array}{l} BPS:equa_{\mathrm{it}}=\alpha +\mathrm{wBPS}:equa_{\mathrm{it}-j}+\beta IE_{\mathrm{it}}\\ \;+\gamma DE_{\mathrm{it}}*IE_{\mathrm{it}}+\delta lnX_{\mathrm{it}}+\varepsilon_{i} \end{array}$$

The idea of the mechanism test model is that after adding the interaction term, as can be seen from Equation (2). The positive and negative values of model coefficients w , γ , and δ as well as the significance of coefficients are analyzed again, with the ultimate goal of judging whether the development of institutional environment can significantly adjust the influence of digital economy on the equalization of public service.

### 3.2 Variables

#### 3.2.1 Explained variable

The EPS_Theil variable, pivotal for evaluating disparities in public service provision across China’s regions, is underpinned by theories from public administration and economics, emphasizing the importance of equal access to public services for social equity and economic efficiency (See Appendix 1 for a detailed overview of the indicator system) (28–30). The methodology for measuring EPS_Theil, specifically the adoption of the Theil Index, is supported by its established use in assessing income inequality and its adaptability for evaluating service provision disparities (31). This approach, detailed in Appendix 4, allows for a nuanced analysis of regional equalization in public services.

#### 3.2.2 Explanatory variable

The entropy method is utilized to measure the development level of the digital economy, as thoroughly evidenced in literature exploring the socio-economic effects of digitalization (12, 32–34). This method’s selection is justified by its efficacy in objectively determining the weights of indicators within the digital economy index system for China, elaborated in Appendix 2. Its aptitude for handling data variability and assigning unbiased importance to indicators makes it particularly appropriate for gauging the advancements in digital technology and their contributions to enhancing the efficiency, accessibility, and responsiveness of public services. The suitability of the entropy method for this analysis is reinforced by literature (35), which highlights its precision in quantifying the nuanced dimensions of digital economy growth, thereby providing a rational and evidence-based foundation for the index system’s formulation.

#### 3.2.3 Mechanism variable

The inclusion of the institutional environment as a mechanism variable draws from institutional theory’s assertion that organizational outcomes are significantly influenced by the surrounding regulatory, normative, and cognitive structures (36). The nuanced measurement of the institutional environment through Fan Gang’s marketization index’s sub-indices, detailed in Appendix 5, aligns with research highlighting the role of institutional factors in economic development (10, 37). This variable facilitates exploration of how institutional contexts mediate the digital economy’s effect on public service equalization.

#### 3.2.4 Control variables

The selection of control variables, such as economic openness (DEO), government intervention (GIL) (38), and transfer payment income (TP) (39), is grounded in economic development and public policy literature (40, 41). These variables, chosen to reflect the broader economic, governmental, and fiscal dimensions influencing public service provision, are critical for constructing a comprehensive analytical framework to investigate the research questions posed by this study.

#### 3.2.5 Instrumental variable

To mitigate endogeneity, this analysis introduces an instrumental variable (DE_IV) for the principal explanatory factor, inspired by methodologies outlined in existing literature. This innovative approach utilizes historical data on post and telecommunications from various regions as indicators of the levels of digital economy development. Following the precedent set by Zhao Tao, data from 31 provinces and cities in 1984 were selected, based on the rationale that traditional communication infrastructures, though foundational to the modern digital economy, have been systematically replaced by advances in digital technology, thereby meeting the criteria for instrument exclusivity (39). This study incorporates a time-varying element, specifically the lagged number of national Internet users, to enhance the robustness of the instrumental variable (42). The combination of these elements, leveraging historical benchmarks and contemporary usage metrics, constructs a robust instrumental variable that adheres to the rigorous standards for addressing endogeneity as demonstrated in the referenced studies.

### 3.3 Data sources

The data utilized in this study are primarily sourced from a range of authoritative and comprehensive repositories. These include the China Statistical Yearbook, China Social Statistics Yearbook, China Urban Yearbook, China Fiscal Yearbook, China Environmental Statistics Yearbook, China Information Technology Yearbook, China Population and Employment Statistical Yearbook, China Internet Development Statistical Report, and China Education Expenditure Statistical Yearbook, covering the period of 2009–2018. Additionally, statistical bulletins on national economic and social development from provinces, autonomous regions, and municipalities directly under the Central Government have been incorporated. Other sources comprise the Enterprise Research Data—Digital Economy Industry Database, the CEIC China Economic Database, the CNNIC China Internet Network Information Center, and the Peking University Digital Financial Inclusion Index.

## 4 Results

### 4.1 Descriptive statistics

Table 1 offers a comprehensive look at the evolution of China’s digital economy alongside the equalization in the provision of basic public services from 2009 to 2018. The data are stratified by region—Eastern, Central, and Western—highlighting regional disparities and progress over time. The EPS_Theil index is utilized to measure the degree of equalization in public services, reflecting the disparities within and across these regions. Concurrently, the Digital Economy (DE) index captures the growth and penetration of digital technologies and their economic impact. This juxtaposition provides valuable insights into the relationship between digital advancement and public service provision, underscoring the dual effects of digitalization. Through a detailed year-by-year and region-by-region analysis, Table 1 lays the groundwork for understanding the complex dynamics at play between technological progress and social equity in the realm of public services.

**Table 1:** Development of China’s digital economy and the level of equalization in basic public service provision.

Index	EPS_Theil				DE			
Region time	Total	Eastern region	Central region	Western region	Total	Eastern region	Central region	Western region
2009	0.0408	0.0430	0.0167	0.0106	0.0460	0.0610	0.0397	0.0365
2010	0.0375	0.0423	0.0159	0.0110	0.0489	0.0669	0.0400	0.0384
2011	0.0337	0.0346	0.0239	0.0054	0.0564	0.0836	0.0452	0.0390
2012	0.0321	0.0330	0.0170	0.0109	0.0697	0.1186	0.0468	0.0401
2013	0.0319	0.0302	0.0169	0.0098	0.0784	0.1339	0.0527	0.0447
2014	0.0300	0.0272	0.0180	0.0104	0.0833	0.1441	0.0560	0.0458
2015	0.0247	0.0201	0.0186	0.0088	0.0982	0.1718	0.0680	0.0509
2016	0.0265	0.0193	0.0186	0.0071	0.1099	0.1999	0.0720	0.0528
2017	0.0284	0.0183	0.0243	0.0068	0.1211	0.2205	0.0740	0.0614
2018	0.0293	0.0228	0.0217	0.0083	0.1484	0.2706	0.0947	0.0721

**Figure 1:**
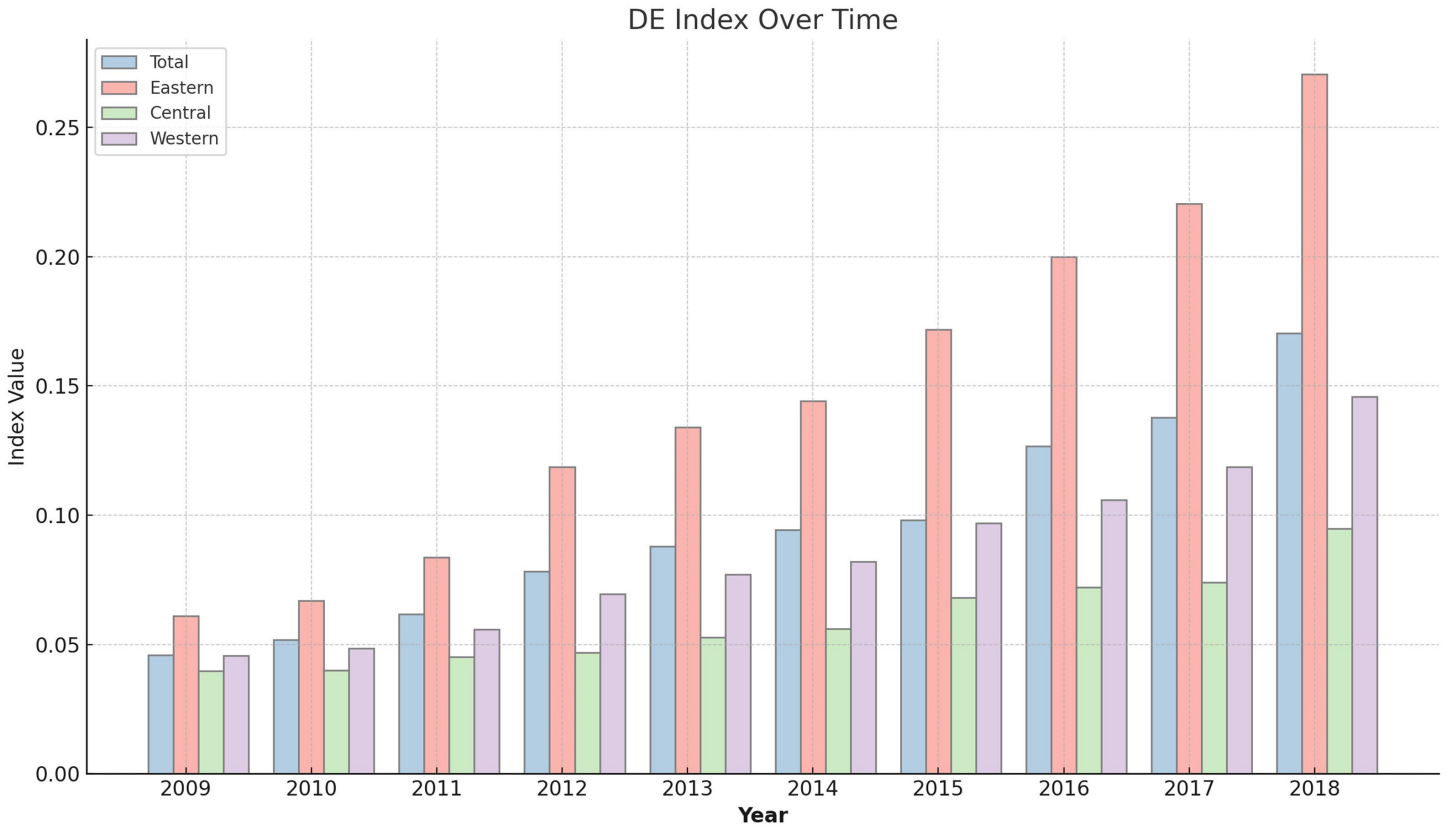
The development level of China’s digital economy.

Figure 1 visualizes the trajectory of China’s digital economy’s development over a decade, encapsulating the rapid growth and transformation within the digital sector. This illustration not only showcases the increasing trend in the digital economy index across all regions but also provides a visual representation of regional variations in digital growth. By mapping out the progression from 2009 to 2018, Figure 1 elucidates the pace and scale of digitalization across China, offering a clear depiction of the country’s commitment to integrating digital technologies within its economic framework.

**Figure 2:**
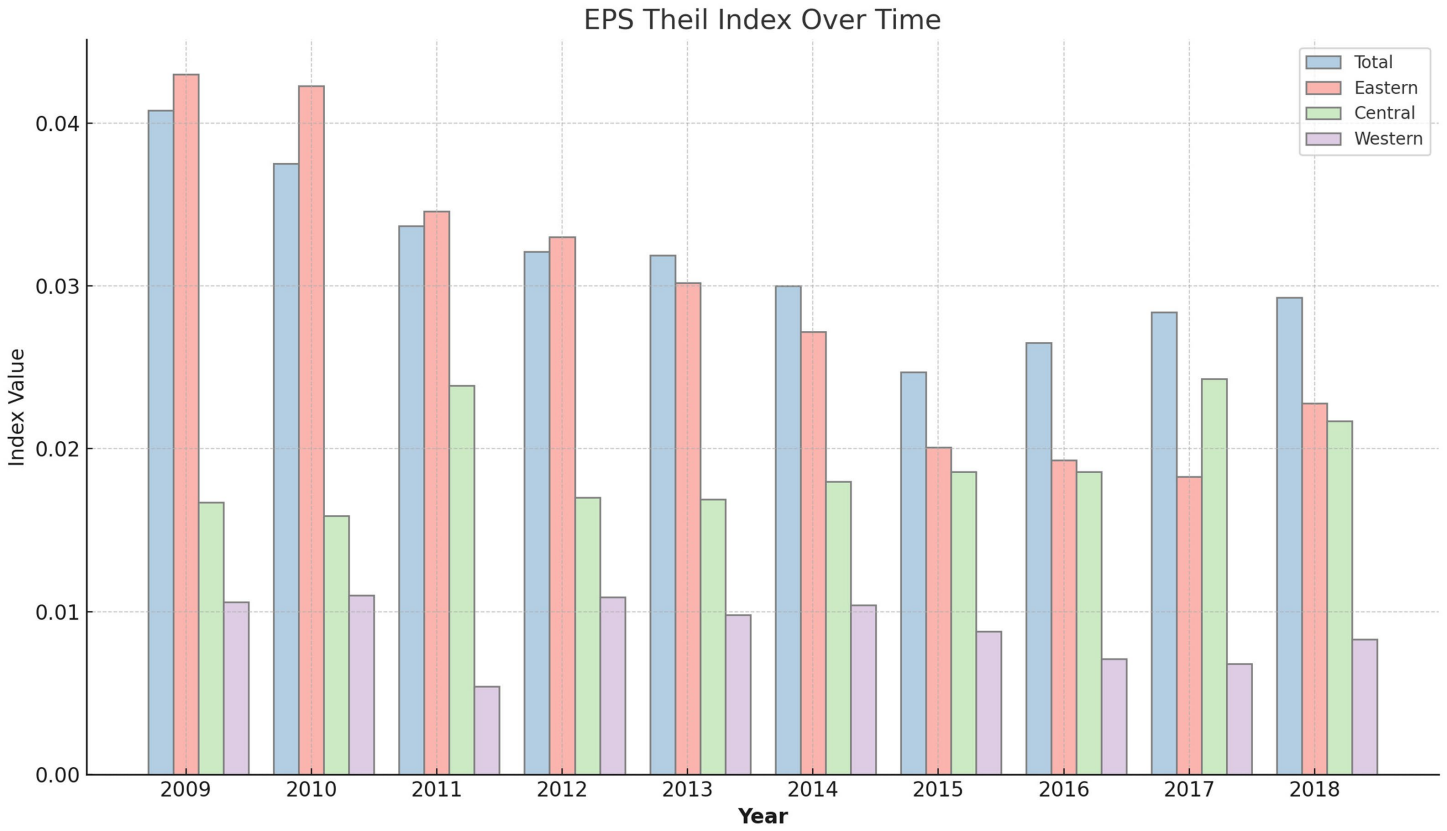
Theil index of the supply of basic public services.

Figure 2 presents the trend in the EPS_Theil index, which quantifies the level of equalization in basic public service provision across China’s diverse regions. This figure brings to light the fluctuations in service provision equalization over time, highlighting both the challenges and achievements in narrowing regional disparities.

The results of the descriptive statistics of the variables in which the development of digital economy in the eastern and western provinces affects the equalization of the supply of basic public services can be seen in Table 2. The eastern region contains a total of 11 provinces, the central region contains a total of eight provinces, and the western region contains 12 provinces. Therefore, the sample size of the eastern and western provinces is 230.

**Table 2:** Descriptive statistics of variables in eastern and western regions.

Variable	Obs	Mean	Std. Dev.	Min	Max
EPS_Theil	230	0.108	0.012	0.093	0.156
DE	230	0.095	0.101	0.023	0.813
DE_IV	230	15.239	1.029	11.907	16.710
DEO	230	0.334	0.355	0.017	1.548
GIL	230	0.332	0.343	0.017	1.548
LnTP	230	16.331	0.614	14.815	17.69
IE	230	0.541	1.141	0.060	8.472
IE*DE	230	0.040	0.090	0.004	1.021

### 4.2 Model specification tests

To rigorously verify the presence of multicollinearity among variables, the variance inflation factor (VIF) was calculated. The results indicate that the highest VIF for models 1–6 is 4.73. Therefore, it can be concluded that models 1–6 do not exhibit severe multicollinearity, which should not impact the accuracy of the empirical results presented in this paper.

After establishing the absence of significant multicollinearity, the choice of panel model was further considered. For models 1–6, the test statistics are not significant, indicating that there is no issue of serial correlation in the error terms of the level equations. Subsequently, the over-identification problem of instrumental variables was assessed, primarily through the Sargan test and the Hansen test, with the results of the Hansen test generally considered more robust. Therefore, this paper reports the results of the Hansen test. The nonsignificant results of the Hansen test indicate the effectiveness of the chosen instrumental variables, justifying the use of the System GMM model for estimation (Table 3).

**Table 3:** Results of model specification tests.

Test	Model-1	Model-2	Model-3	Model-4	Model-5	Model-6
Wald $-\chi^{2}$	85921.79 (0.000)	254562.10 (0.000)	80917.86(0.000)	464360.26 (0.000)	200630.13 (0.000)	721923.06 (0.000)
Hansen	20.43 (0.990)	22.78 (0.995)	21.52 (0.997)	22.93 (0.995)	22.64 (0.995)	22.84 (0.927)
AR (1)	−2.59 (0.010)	−2.60 (0.000)	−2.61 (0.009)	−2.54 (0.000)	−2.67 (0.000)	−2.67 (0.000)
AR (2)	0.64 (0.522)	0.86 (0.414)	0.63 (0.526)	0.66 (0.504)	−1.88 (0.602)	−1.86 (0.664)

p values in parentheses.

### 4.3 Benchmark model and endogeneity test results

In order to verify the impact of the development of digital economy on the equalization degree of the supply of basic public services, an empirical modeling was conducted for the two. In this study, the benchmark model is denoted as model 1, and the degree of public service equalization that lags the second stage is denoted as model 2.

Existing research exploring the disparity between digital economy development and regional basic public service provision remains limited in adequately addressing endogeneity issues. This deficiency is largely attributed to the scarcity of empirical studies that examine regional public service supply disparities from a digital economy development standpoint, compounded by the lack of a universal instrumental variable. Consequently, potential reverse causality could skew the estimation outcomes of public service supply equalization in the context of digital economy development, with regional imbalances in basic public service provision reciprocally influencing regional inequality and subsequent digital economy progression. To rectify this, this paper employs an instrumental variable approach to test for endogeneity, substituting the original explanatory variable with DE_IV, an instrumental variable reflecting the digital economy’s development level (Model 3). In Model 4, the lagged second phase of public service equalization is employed. The estimated results of the model can be seen in Table 4.

**Table 4:** Model estimation results.

Variable	Model-1 EPS_Theil	Model-2 EPS_Theil	Model-3 EPS_Theil	Model-4 EPS_Theil
L.EPS_Theil	0.5730^***^ (2.62)		0.7404^***^ (20.58)	
		
L2.EPS_Theil		0.8636^***^ (54.32)		0.5209^***^ (19.71)
		
DE	0.1837^**^ (2.45)	0.0326^**^ (2.33)		
		
DE_IV			0.0482^***^ (2.92)	0.0445^***^ (4.09)
		
DEO	−0.0136 (−0.18)	0.0093^***^ (3.87)	0.0050^***^ (2.87)	0.0041^*^ (1.65)

GIL	0.5062 (1.48)	−0.0017 (−0.56)	0.0008 (0.26)	0.0000 (0.00)

LnTP	−0.0771^**^ (−2.06)	0.0054^***^ (8.76)	0.0042^***^ (5.96)	0.0045^***^ (6.02)

_cons	−1.6665^*^ (−1.69)	−0.0505^***^ (−5.46)	−0.0377^***^ (−3.14)	−0.0480^***^ (−5.31)

*N*	207	184	207	184

The numbers in brackets are *t* values, and ^***^, ^**^, and ^*^ are significant levels of 1, 5, and 10%, respectively.

Based on the analysis of panel data from 31 provinces and cities in China spanning from 2009 to 2018, this study leverages a system GMM dynamic panel model to assess the effect of digital economy development on the equalization of basic public service provision. Two main findings emerge from the study:

Firstly, the growth of the digital economy has a substantial intensifying impact on disparities in basic public service provision, which ultimately inhibits equalization. Despite the digital economy’s broad contribution to enhancing the quality of basic public services in China, it opposes efforts to promote equality, thereby undermining societal fairness and stability. Secondly, the system GMM model’s estimations reveal that irrespective of a 1-year or 2-year lag, disparities in basic public service provision have a significant positive influence on current inequalities. This result underscores the self-reinforcing nature of regional supply equalization and its dependence on previous service levels.

Then the endogeneity test results, characterized by consistency between the instrumental variable DE_IV estimates and the benchmark model, reaffirm the conclusion’s reliability that digital economy development exacerbates regional supply disparities, thereby impeding supply equalization. These results further substantiate the robustness of our previous findings.

### 4.4 Mechanism analysis

This paper introduces the interaction term (D_E*I_E) between the development of digital economy and institutional environment, and builds a test model on this basis to empirically test the regulatory role of institutional environment on the impact of the development of digital economy on the equalization degree of the supply of basic public services. The specific model estimation results are shown in Table 5.

**Table 5:** Mechanism analysis result.

Variable	Model-5 EPS_Theil	Model-6 EPS_Theil
L.EPS_Theil	0.9009^***^ (26.28)	0.8335^***^ (22.78)

DE	0.0065^*^ (1.94)	
	
DE_IV		0.0707^***^ (3.74)
	
IE	0.0041^***^ (7.00)	0.0124^***^ (6.34)

IE*DE	−0.0343^***^ (−8.12)	−0.1954^***^ (−5.80)

DEO	0.0025^***^ (4.46)	0.0109^***^ (2.90)

GIL	−0.0018^**^ (−2.18)	−0.0041^***^ (−2.64)

LnTP	0.0011^***^ (4.03)	0.0051^***^ (5.59)

_cons	−0.0178^***^ (−3.96)	−0.0693^***^ (−4.97)

N	207	207

The numbers in brackets are *t* values, and ^***^, ^**^, and ^*^ are significant levels of 1, 5, and 10%, respectively.

Table 5 indicates that the primary explanatory variable, the digital economy development index, is significant at the 1% level. Furthermore, the interaction term between digital economy development and the institutional environment is negatively significant at the 1% level. This suggests a substantial moderating effect of the institutional environment on the supply gap of regional basic public services, exacerbated by digital economy development. Hence, bolstering the institutional environment is critical in mitigating the adverse impacts of digital economy development on supply equalization.

## 5 Discussion

### 5.1 The paradox of digital economy and equalization degree of public service provision

This study’s utilization of a system GMM dynamic panel model, detailed in Table 4, effectively addresses the intricate relationship between digital economy development and the equalization of public service provision. The methodological rigor, enhanced by the employment of DE_IV as an instrumental variable, marks a pivotal step in overcoming endogeneity issues, often overlooked in previous research.

The findings, rooted in the analysis of panel data spanning from 2009 to 2018 across 31 provinces and cities in China, highlight a significant paradox. Despite the digital economy’s potential to enhance the quality of basic public services (43, 44)—a benefit widely anticipated and supported by the literature, including contributions from Brynjolfsson and McAfee (33) and Castells (32)—it simultaneously exacerbates regional disparities. This dichotomy aligns with the concerns of Norris (45) and Van Dijk (46) regarding the digital divide, suggesting that without careful policy consideration, digital advancements could widen existing inequalities.

Table 4‘s results substantiate the dual nature of digital economy growth: its broad contributions to service quality improvement contrast with its role in intensifying disparities. This observation is crucial in the context of China’s digital transformation ambitions, where the potential for digitalization to promote equality faces challenges from entrenched regional and socio-economic disparities. The study’s empirical evidence, showcasing the self-reinforcing nature of regional supply equalization and its dependency on historical levels of service provision, underscores the complexities of achieving equitable public services in the digital age.

In summary, the discussion, enriched by references and findings from Table 4, underlines the importance of a nuanced approach to digital economy development that considers both its potential benefits and its challenges. The study calls for an integrated policy strategy that leverages digital innovations while ensuring that their benefits are equitably shared, thereby contributing to the broader goals of societal fairness and stability. This balanced perspective is essential for harnessing the digital economy as a catalyst for inclusive public service provision in China and beyond.

### 5.2 The pivotal role of the institutional environment in moderating the digital economy’s impact

The findings from Table 5, delineating the interaction between digital economy development and the institutional environment, significantly enrich the discourse on digital transformation’s impact on public service provision. This analysis underscores a critical insight: while the digital economy harbors transformative potential for public services, its benefits are intricately tied to the strength and adaptability of institutional frameworks. The introduction of the interaction term (D_E*I_E) provides a nuanced lens through which to examine the regulatory role of these frameworks, revealing that a conducive institutional environment is paramount for mitigating the adverse impacts of digital economy development on service provision equalization.

The negative significance of the interaction term between digital economy development and the institutional environment highlights the substantial moderating effect institutions have on the relationship between digital advancements and the equalization of public services. This finding aligns with the assertions of North (47), who emphasizes the foundational role of institutions in shaping economic outcomes, and Acemoglu and Robinson (48, 49), who argue that inclusive institutions are crucial for leveraging technological advancements for broad-based growth and equality. The study’s evidence suggests that without a supportive institutional environment, the gap in the provision of basic public services across regions could widen, reinforcing the need for robust governance structures that can harness the digital economy’s growth for societal benefit.

In summary, the insights gleaned from this study advocate for a policy approach that transcends mere digital infrastructure development, emphasizing the need for institutional reforms aimed at ensuring that digital economy benefits are equitably distributed. This dual focus on technological innovation and institutional enhancement is essential for realizing the full potential of digital transformations in public service provision, contributing to a more inclusive and equitable society. Such an approach resonates with the broader policy recommendations of Margetts and Dunleavy (17, 50), who call for integrated strategies that combine technological advancements with institutional capacity building to improve public sector efficiency and accessibility.

### 5.3 Policy implication

In crafting policies that align digital advancements with the equitable provision of public services, it is imperative to adopt a nuanced approach that is informed by a confluence of theoretical insights and empirical evidence. The transformative potential of digital technologies in public administration, as articulated by Margetts and Dunleavy (17), underscores the necessity of integrating digital solutions to enhance efficiency, transparency, and citizen engagement. However, this potential can only be fully realized when accompanied by concerted efforts to address the digital divide, a critical barrier to universal access and utilization of digital services. Warschauer’s (51) exploration of the digital divide elucidates the imperative for policies that not only extend digital infrastructure but also foster digital literacy and inclusivity, ensuring that technological advancements do not exacerbate existing social inequalities.

The role of adaptive governance structures in navigating the challenges and opportunities presented by the digital transformation is further emphasized by Heeks and Bailur (52). Their analysis highlights the importance of evolving institutional frameworks that are responsive to technological innovation, advocating for regulatory agility and policy flexibility. This perspective is complemented by Ostrom’s (53) advocacy for polycentric governance models, which posits that diverse and decentralized governance structures can enhance the effectiveness and equity of public service delivery.

Moreover, the significance of collaborative governance in devising inclusive and sustainable digital policies is illuminated by the work of Ansell and Gash (54). Their research on collaborative governance models demonstrates the value of multi-stakeholder engagement, suggesting that incorporating a wide range of perspectives can lead to more innovative and equitable solutions.

Synthesizing these insights, it becomes evident that an integrated policy strategy is essential for leveraging digital technologies in a manner that promotes public welfare while mitigating the risks of increased social disparities. Such a strategy should encompass the strengthening of governance frameworks, the bridging of the digital divide through targeted interventions, and the fostering of collaborative ecosystems that engage all sectors of society.

## 6 Conclusion

This study has ventured into the complex interplay between the digital economy and the provision of equitable public services, illuminating a domain that remains underexplored within the vast expanse of contemporary research. By employing a system Generalized Method of Moments (GMM) dynamic panel model, this research has unraveled the paradoxical nature of the digital economy: its capacity to enhance public service quality simultaneously magnifies regional disparities, thereby shedding light on a layer of complexity that has not been adequately addressed in prior studies.

The findings underscore the persistent nature of inequality in public service provision, echoing and expanding upon Piketty’s seminal work on cumulative inequality. This deepens the discourse on socioeconomic disparities, highlighting the ongoing challenge of achieving equitable public service provision in an era of rapid digital transformation. A significant contribution of this research lies in its exploration of how the institutional environment can act as a lever to counteract the adverse effects of the digital economy, signaling a critical avenue for policy interventions aimed at fostering robust institutional frameworks in the digital era.

In recognizing the limitations of the current study, future research avenues are already being charted with an emphasis on broadening the analytical scope. A key area identified for expansion is the application of heterogeneity analysis to dissect the nuanced impacts of the digital economy on public service provision across different regions, demographic groups, and socioeconomic strata. This methodological enhancement aims to unearth the varied effects of digital transformation, providing a more detailed mapping of who benefits and who may be left behind in the digital era.

In sum, this research marks a foundational step toward a more comprehensive understanding of the digital economy’s implications for public service equity. It calls for a continued scholarly engagement with the challenges and opportunities presented by digital transformation, urging for innovative policy solutions that ensure the benefits of the digital economy are equitably distributed. As we move forward, the insights gleaned from this study serve as a crucial groundwork for future research endeavors aimed at navigating the complexities of achieving social equity in the digital age.

## Data availability statement

The raw data supporting the conclusions of this article will be made available by the authors, without undue reservation.

## Author contributions

YL: Conceptualization, Data curation, Formal analysis, Investigation, Writing – original draft. JX: Conceptualization, Formal analysis, Methodology, Project administration, Resources, Visualization, Writing – review & editing. XM: Methodology, Funding acquisition, Writing – original draft. XW: Funding acquisition, Investigation, Project administration, Resources, Validation, Visualization, Writing – review & editing.
