# Supplementary material for: Digital economy and institutional dynamics: striving for equitable public service in a digitally transformed era
Source: Front Public Health. 2024 Mar 21;12:1330044. doi: 10.3389/fpubh.2024.1330044 (PMC10993998; doi:10.3389/fpubh.2024.1330044)
Supplement: Supplementary file 1 [file Table_1.docx]

**Appendix**

Appendix Table 1 Evaluation index system of basic public service supply level

| First-grade index | Second-grade index | Third-grade index | Symbol |
| --- | --- | --- | --- |
| Basic public service supply level | basic public education | Per capita years of education (years) X1 | + |
|  |  | The proportion of illiterate and semi-literate population aged 15 and over (%)X2 | - |
|  |  | Enrollment rate of school-age children (%)X3 | + |
|  |  | Primary school teacher-student ratio (%) X4 | + |
|  |  | Junior high school teacher-student ratio (%) X5 | + |
|  |  | Normal high school teacher-student ratio (%)X6 | + |
|  | Basic Labor Employment Entrepreneurship | Proportion of employed persons in urban units (%) X7 | + |
|  |  | Urban registered unemployment rate (%)X8 | - |
|  |  | Average salary of employed persons in urban units (yuan) X9 | + |
|  |  | Entrepreneurship service number (person) X10 | + |
|  | basic social insurance | Number of people participating in work-related injury insurance at the end of the year (10,000 people) X11 | + |
|  |  | Unemployment insurance coverage (%) X12 | + |
|  |  | Coverage rate of urban basic endowment insurance at the end of the year (%)) X13 | + |
|  |  | Coverage rate of urban employees' basic medical insurance at the end of the year (%) X14 | + |
|  |  | Number of people participating in maternity insurance at the end of the year (10,000 people) X15 | + |
|  | basic health care | Number of doctors per 10,000 people (person) X16 | + |
|  |  | Number of health technicians per 10,000 people (person) X17 | + |
|  |  | Number of beds per 10,000 people (piece) X18 | + |
|  |  | Emergency case fatality rate (%)X19 | - |
|  |  | Diagnosis and treatment visits per capita in medical and health institutions (person visits) X20 | + |
|  |  | Number of medical and health institutions per unit area (pieces) X21 | + |
|  |  | Number of Centers for Disease Control and Prevention (units) X22 | + |
|  | basic social services | Orphan subsidy level (yuan/year/person) X23 | + |
|  |  | Number of Assistant Social Workers of Community Neighborhood Committees (person) X24 | + |
|  |  | Nursing expenses for scattered resettlement of disabled soldiers within the civil affairs budget (10,000 yuan) X25 | + |
|  |  | Regular quantitative subsidy level for retired soldiers in the countryside (yuan/person/year) X26 | + |
|  |  | Child dependency ratio (child burden factor) (%) X27 | - |
|  |  | Subsidy level of senior affairs institutions (yuan/person/year) X28 | + |
|  |  | Number of schools for the elderly (units) X29 | + |
|  |  | Overall expenditure of welfare budget funds for the elderly (10,000 yuan) X30 | + |
|  | basic housing security | Building area under construction (10,000 square meters) X31 | + |
|  |  | Completed housing area (10,000 square meters) X32 | + |
|  |  | Green coverage rate of built-up area (%)X33 | + |
|  |  | Water penetration rate (%)X34 | + |
|  |  | Gas penetration rate (%)X35 | + |
|  |  | Sanitary toilet penetration rate (%)X36 | + |
|  | Basic public cultural and sports services | TV population coverage (%)X37 | + |
|  |  | Broadcast population coverage (%)X38 | + |
|  |  | Construction area of gymnasium owned by 10,000 people (square meter) X39 | + |
|  |  | Area of public library owned by 10,000 people (square meter) X40 | + |
|  |  | Building area of mass cultural facilities owned by 10,000 people (square meter) X41 | + |
|  |  | Number of reading room seats per 10,000 people (number) X42 | + |
|  |  | Per capita cultural undertaking expenses (yuan) X43 | + |
|  | Basic public services for the disabled | Rescue institutions for the needy people this year (10,000 people) X44 | + |
|  |  | Number of employed persons with disabilities (10,000 people) X45 | + |
|  |  | Number of disabled persons with minimum living guarantee for urban residents (person) X46 | + |
|  |  | Number of disabled persons with minimum living guarantee in rural areas (person) X47 | + |

Appendix Table 2 Evaluation index system of digital economy development level

| First-grade index | Second-grade index | Third-grade index | Symbol |
| --- | --- | --- | --- |
| Digital economy development level | Digital industry | Proportion of employment in urban units in information transmission, computer services and software industries (%)X1 | + |
|  |  | Software business revenue (Billion)X2 | + |
|  |  | Proportion of information transmission, computer services and software industries in the total fixed assets of the society(%)X3 | + |
|  |  | Digital HP financial index(/)X4 | + |
|  | Digital innovation | Number of 5G industry authorized patents (PCS )X5 | + |
|  |  | Number of industrial internet authorized patents(PCS )X6 | + |
|  |  | Number of e-commerce authorized patents(PCS )X7 | + |
|  | Digital users | Popularization rate of mobile telephones（PCS/one hundred）X8 | + |
|  |  | Total telecommunications business（Billion ）X9 | + |
|  |  | Per capita internet broadband access users(PCS )X10 | + |
|  | Digital platform | Number of domain names（Ten thousand/PCS）X11 | + |
|  |  | Number of internet users（Ten thousand person(Ten thousand /person）X12 | + |
|  |  | Number of websites（Ten thousand /PCS）X13 | + |

Appendix Table 3 Division of eastern, central and western China

| Three regional economic belt division | |
| --- | --- |
| Eastern region | Beijing, Tianjin, Hebei, Liaoning, Shanghai, Jiangsu, Zhejiang, Fujian, Shandong, Guangdong and Hainan |
| Central region | Shanxi, Jilin, Heilongjiang, Anhui, Jiangxi, Henan, Hubei and Hunan |
| Western region | Inner Mongolia, Guangxi, Chongqing, Sichuan, Guizhou, Yunnan, Tibet, Shaanxi, Gansu, Qinghai, Ningxia and Xinjiang. |

Appendix 4: Formula of Theil Index

If the sample of n individuals is divided into k groups, then each group is:

$g_{k}(k=1,2,\cdots,K)$ (1)

The number of individuals in group $k$ $g_{k}$ is $n_{k}$, then there is $\sum_{k=1}^{K} n_{k}=n$.

Where *y_i_* represents the supply level of basic public services of an individual i, and *y_k_* is the sum of supply levels of a group k. Theil index can be decomposed into inter-regional gap index and intra-regional gap index, namely T_b_ and T_w_, and the decomposition formulas are shown in (2), (3) and (4).

$T=T_{b}+T_{w}=\sum_{k=1}^{K} y_{k}log\frac{y_{k}}{n_{k}/n}+\sum_{k=1}^{K} y_{k}\left( \sum_{i\in g_{k}} \frac{y_{i}}{y_{k}}log\frac{y_{i}/y_{k}}{1/n_{k}} \right)$ (2)

The formula for the inter-regional gap $T_{b}$ of public service supply is as follows:

$T_{b}=\sum_{k=1}^{K} y_{k}log\frac{y_{k}}{n_{k}/n}$ (3)

The formula of intra-regional gap $T_{w}$ of public service supply is as follows:

$T_{w}=\sum_{k=1}^{K} y_{k}\left( \sum_{i\in g_{k}} \frac{y_{t}}{y_{k}}log\frac{y_{t}/y_{k}}{1/n_{k}} \right)$ (4)

Appendix 5: Formula of Institutional Environment

$\Delta{ReformSub-index}_{\mathrm{jrt}}=\left| \left( {ReformSub-index}_{\mathrm{jrt}} \right) \right.-\left( {ReformSub- \mathrm{index}}_{jrt-1} \right)$ (5)

$\Delta R_{jtt}=\frac{\Delta ReformSub-index_{jtt}}{\sum_{j=1}^{5} Re\text{ form }Sub-index_{jr}}$ (6)

$IE_{\mathrm{it}}=1/{\mathrm{Max}_{t}-\sum_{j=1}^{5} \Delta R_{\mathrm{jt}}\times ln\left( \frac{1}{\Delta R_{\mathrm{jit}}} \right)}$ (7)

The $\Delta$ ReformSub-index${}_{jn}$ in formula (5) is the absolute value of the change of $sub-index_{jn}$ from the previous year to the current year in 31 provinces and cities $(r)$ and 5 sub-indicators $(j)$. Then calculate the change relationship between each sub-index and the five dimensions. Finally, formula $\sum_{j=1}^{5} \Delta R_{\mathrm{jt}}\times ln\left( \frac{1}{\Delta R_{\mathrm{jit}}} \right)$ is used to calculate the change rate of the five sub-indicators, and $IE_{\mathrm{it}}$ in formula (7) is the institutional environment.
